# A Rare Case of Benign Phyllodes Tumor of the Breast Responding to Anastrozole

**DOI:** 10.7759/cureus.77277

**Published:** 2025-01-11

**Authors:** Patrícia R Rodrigues, Diana Mata, José Brito-da-Silva, Carolina P Duque, Ana Ferreira

**Affiliations:** 1 Medical Oncology, Instituto Português de Oncologia do Porto Francisco Gentil, Centro Hospitalar de Lisboa Central (EPE), Porto, PRT; 2 Pathology, Instituto Português de Oncologia do Porto Francisco Gentil, Centro Hospitalar de Lisboa Central (EPE), Porto, PRT

**Keywords:** anastrozole, colloid carcinoma, fibroepithelial lesion, phyllodes tumour, primary breast malignancy

## Abstract

A phyllodes tumor is a rare fibroepithelial breast tumor that can be benign, borderline, or malignant. Its diagnosis is challenging because it shares characteristics with other lesions. The treatment is primarily surgical, with no established role for chemotherapy and limited evidence supporting radiotherapy. There is no evidence of phyllodes tumors expressing endocrine receptors, nor is there an established role for endocrine therapy in treating these tumors. We present a case of a large phyllodes tumor that showed a clinical response to treatment with anastrozole.

## Introduction

Phyllodes tumor is a rare fibroepithelial breast neoplasm, usually found in women with an age range from 40 to 50 years old, and represents less than 1% of all breast tumors [1,2]. Histologically, they have a stromal origin and epithelial elements, such as fibroadenomas [1,3,4]. The World Health Organization (WHO) classifies these tumors as benign, borderline, or malignant, based on morphological characteristics [1,5]. The most common grade is benign, representing between 60% and 75% of the cases [2]. Treatment primarily relies on surgery, while the roles of chemotherapy and radiotherapy remain unclear and not well-established [1,3]. There is no evidence in the literature supporting endocrine therapy for these types of tumors. We present a case of a benign phyllodes tumor that responded to anastrozole (an aromatase inhibitor), prescribed due to a misdiagnosis of a breast carcinoma. This unexpected clinical response underscores the importance of meticulous pathological assessment in fibroepithelial lesions and raises the possibility that endocrine therapy may have a role in select phyllodes tumor cases. Further investigation is warranted to clarify the mechanisms driving this response and the potential clinical implications for disease management.

## Case presentation

**Figure 1:**
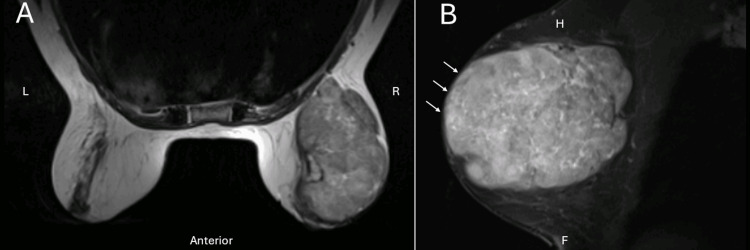
Magnetic resonance imaging of the breast (T2-weighted scan) in axial (A) and sagittal (B) plane showing a lesion occupying almost the entire right breast, with well-defined lobulated contours, measuring 93x71x82 mm and contact with the pectoral muscle and the skin surface (white arrows). F: foot direction; H: head direction; L: left direction; R: right direction

A 64-year-old woman with a history of a right breast lump for 10 years, previously deemed a "benign fibroadenoma" and under surveillance, presented to a consultation due to the perception of an increase in the lump over the previous three months with associated discoloration of the adjacent skin. She had no other noteworthy symptoms. Regarding her family history, the patient’s mother and two maternal aunts had localized breast cancer. Clinical examination confirmed a lump in the upper outer quadrant of the right breast, measuring 8 cm at its largest dimension, hard to the touch, and fixed to the deeper tissue. Blood work did not reveal any abnormalities. A magnetic resonance imaging (MRI) of the breast was performed and showed a lesion occupying almost the entire right breast, with well-defined lobulated contours, measuring 93x71x82 mm, showing a high signal on T2-weighted images, and contacting the pectoral muscle and the skin surface (Figure 1).

**Figure 2:**
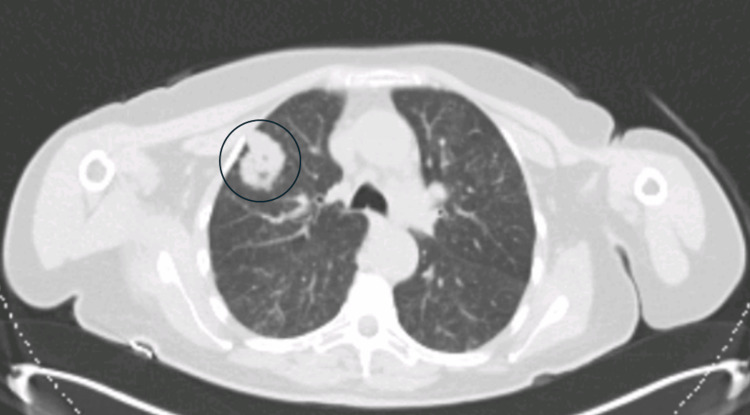
Computer tomography scan of the thorax showing showing in the apical segment of the right upper lobe, a nodule measuring 34 mm (black circle), adjacent to the anterior pleura with a slight retraction.

A computed tomography (CT) scan of the thorax, abdomen, and pelvis revealed a nodule in the apical segment of the right upper lobe, measuring 34 mm in diameter, suspected to be metastasis (Figure 2).

**Figure 3:**
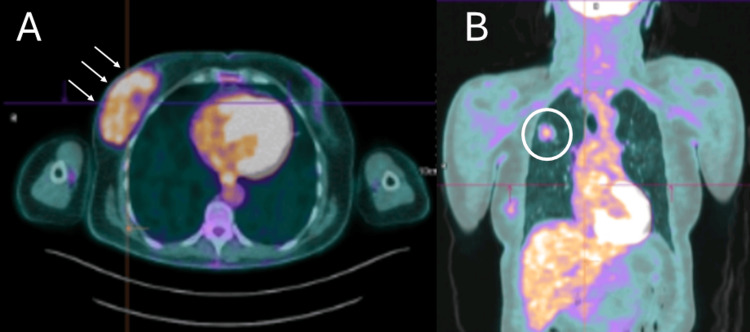
A fluorodeoxyglucose (FDG)-positron emission tomography (PET) scan showing a moderate uptake of 18F-FDG (SUVmax = 3.15) in a large formation in the outer quadrants of the right breast (white arrows), and a moderate uptake of 18F-FDG (SUVmax = 2.35) in a pulmonary lesion of the right upper lobe (white circle).

A fluorodeoxyglucose (FDG) positron emission tomography (PET) scan showed a moderate uptake of 18F-FDG (SUVmax = 3.15) in a large formation centered in the outer quadrants of the right breast, and a moderate uptake of 18F-FDG (SUVmax = 2.35) in the pulmonary lesion of the right upper lobe (Figure 3).

The patient underwent four biopsies (two from the breast tumor and two from the axillary nodes), which revealed fibromatosis and a biphasic proliferative lesion without evidence of malignancy in the breast, with negative nodes. Nevertheless, because of the significant clinical findings (a large breast mass and a suspicious lung nodule), the attending physicians maintained a high suspicion of metastatic breast cancer. Consequently, they initiated palliative endocrine therapy with anastrozole (1 mg once daily) and referred the patient to a comprehensive cancer center in Portugal for further evaluation.

**Figure 4:**
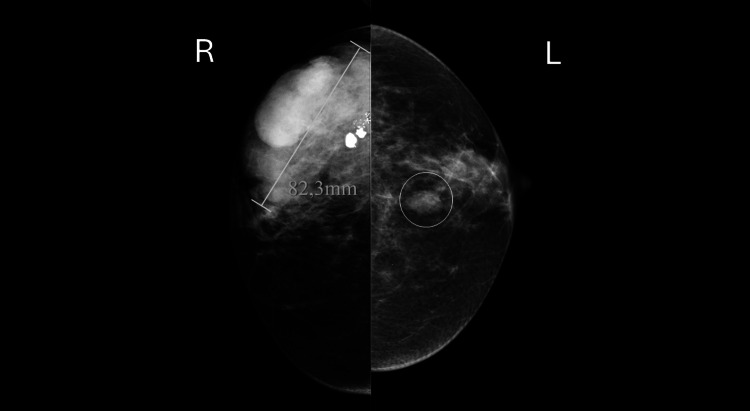
Breast mammography Breasts with a moderately dense pattern. A large, high-density mass with lobulated contours, measuring approximately 8.2 cm, is identified in the upper outer quadrant/transition of the outer quadrants of the right breast, with coarse calcifications at the periphery, corresponding to the previously known neoformative lesion. In the left breast, a medium-density nodule with slightly lobulated margins is seen in the transition of the upper quadrants (circle), which corresponds to a cyst on ultrasound.

After four months of treatment and upon her arrival at our comprehensive cancer center, the patient reported a noticeable improvement in the lump since starting anastrozole. A new mammography was performed, revealing a reduction in the size of the lesion (Figure 4).

**Figure 5:**
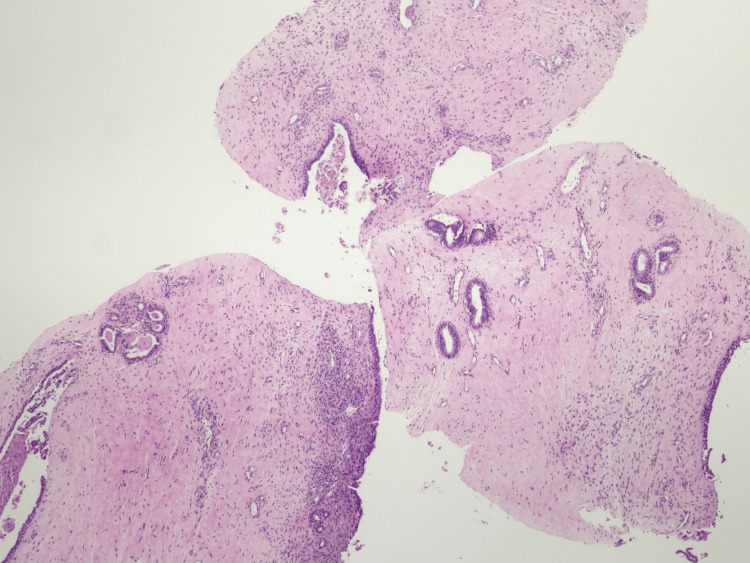
Low magnification of the core biopsy showing a fibroepithelial lesion with expansion of the stroma and low cellularity.

**Figure 6:**
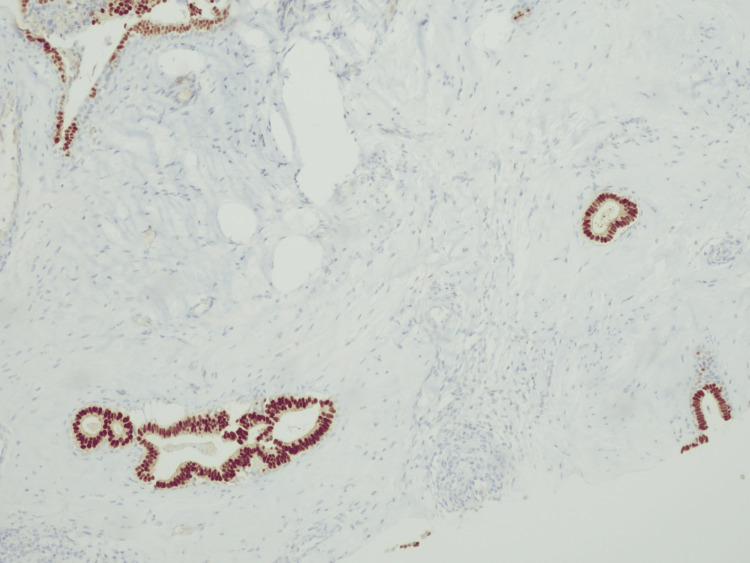
Immunoreactivity for estrogen receptors in the epithelial component detected in the core biopsy.

A new core biopsy was performed, and histological and immunohistochemical examination revealed a fibroepithelial lesion with benign characteristics, favoring the diagnosis of a benign phyllodes tumor (Figure 5). Due to the favorable response to anastrozole, immunohistochemical analysis of endocrine receptors was performed, revealing 90-100% positivity for estrogen receptors in the epithelial component (Figure 6), without expression of progesterone receptors and human epidermal growth factor receptor 2 (HER2). A core biopsy of the lung nodule was also performed and revealed a neoplastic lesion with atypical mucinous cells expressing CK7 and CDX2, without CK20 expression, and with ambiguous expression of TTF1, suggesting a colloid adenocarcinoma of the lung.

**Figure 7:**
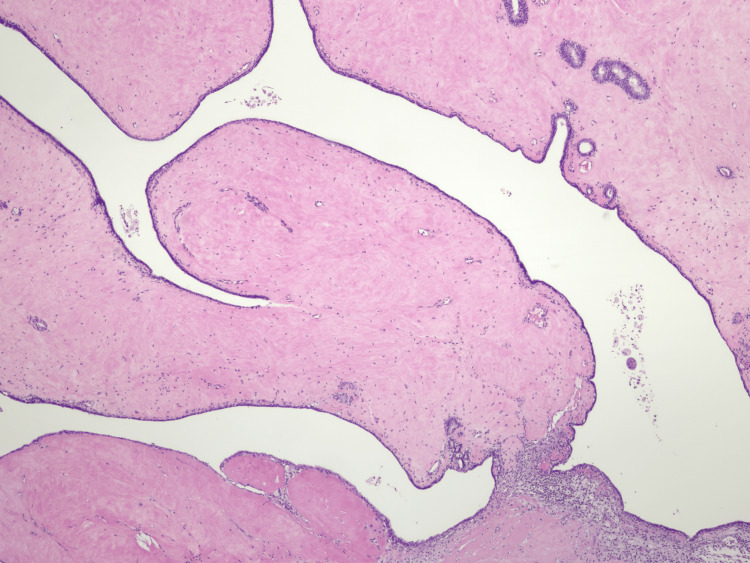
Histologic section showing fronds of homogeneous stroma with low cellularity covered by a thin layer of epithelium, compatible with a phyllodes tumor.

**Figure 8:**
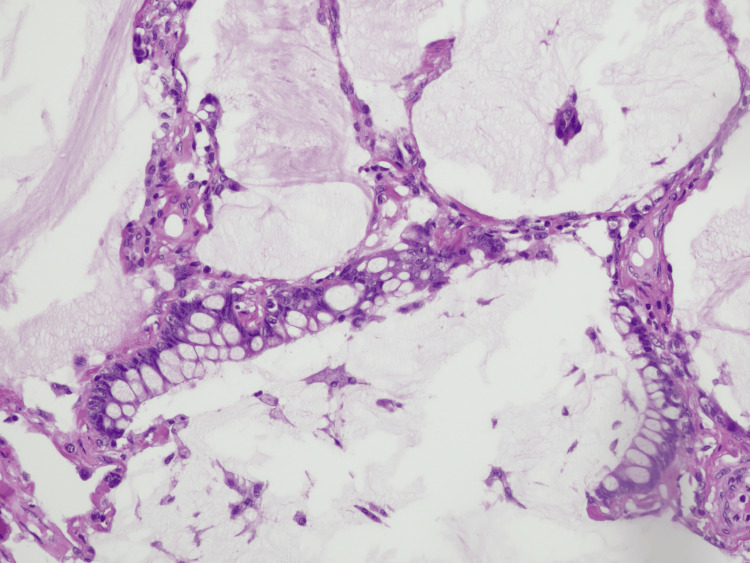
High magnification of a histologic section of the colloid adenocarcinoma of lung showing cuboidal tumor cells with hyperchromatic nuclei lining a mucin pool. Tumor cells markedly distend airspaces and destroy alveolar walls.

Since there was tumor reduction and new imaging evaluation excluded distant metastases, a multidisciplinary team discussion took place, including experts in connective tissue neoplasms and lung cancer. The patient was proposed to stop anastrozole and underwent a right partial mastectomy. The operative specimen revealed a tumor measuring 76x44x46 mm, consistent with a benign phyllodes tumor and without signs of malignancy (Figure 7); there was no evidence of margin involvement (R0 surgery). After this surgery, the patient underwent a right upper lobectomy, which revealed a colloid adenocarcinoma of the lung (Figure 8). The lesion measured 37 mm in dimension, had no lymphovascular invasion, node invasion, or involved margins, and PD-L1 was negative. This lesion was staged as pT2 pN0 cM0, according to the eighth edition of the American Joint Committee on Cancer (AJCC). She was placed under surveillance and has been disease-free for at least eight months.

## Discussion

Phyllodes tumor of the breast is a rare occurrence, with an incidence of 2.1 per million [5]. Clinical presentation is usually a rapidly growing mass, and mammography usually shows a well-circumscribed oval or lobulated mass. The diagnosis is challenging due to the high resemblance of benign phyllodes tumors with benign fibroadenomas, since they share microscopic features [6]. Phyllodes tumors are characterized by a leaflike architecture due to the intracanalicular growth of the hypercellular stroma producing cleft-like spaces [7]. Benign phyllodes tumor has a mildly increased stromal cellularity and mild nuclear atypia, with rare mitoses. These characteristics, especially the increased stromal cellularity, are shared with fibroadenoma, although the leaflike pattern should not appear in fibroadenomas. Another difficulty in the diagnosis is the fact that there may be areas with fibroadenoma-like characteristics in phyllodes tumors. To complicate matters further, there is poor interobserver reproducibility in diagnosing these tumors [7]. Therefore, these tumors are a challenge to diagnose. In this case, the diagnosis was only made after referral to a comprehensive cancer center, highlighting the importance of having an experienced pathologist for diagnosing stromal tumors. Another diagnostic challenge arises from the differential diagnosis on core needle biopsies, where phyllodes tumors can resemble other spindle cell lesions, such as fibromatosis or metaplastic spindle cell carcinoma and even fibroadenoma. For malignant phyllodes tumors, differential diagnoses include metaplastic carcinoma, primary sarcoma, or metastatic sarcoma. Diagnostic challenges with phyllodes tumors are often related to the limited representativeness of biopsy samples, rather than the excised specimen [8].

The primary treatment for this disease is surgery (lumpectomy or mastectomy), without axillary staging since this disease is not known to spread to the lymph nodes [9]. None of the actual recommendations suggest chemotherapy for phyllodes tumors [6]. Adjuvant radiotherapy may be considered in borderline or malignant phyllodes tumor, since it can improve local recurrence rate, although it seems to lack benefit in terms of overall and disease-free survivals [2,10]. Benign phyllodes tumors have a very good prognosis, with a low recurrence rate (10-17% of local recurrence) and almost no specific mortality [6].

To our knowledge, this is the first case report to show a phyllodes tumor responding to endocrine therapy with anastrozole. This drug targets aromatase, an enzyme that converts androgen to estrogen, and is amply used in endocrine receptor-positive breast cancer, since it significantly reduces serum estrogen levels, decreasing the activation of estrogen and progesterone receptors in breast cancer [11]. There is some evidence of endocrine receptor expression in these tumors. Tse et al. [12] reviewed the hormonal receptor expression in 143 phyllodes tumors and found that there was a higher expression of estrogen receptors (58%) and progesterone receptors (74.8%) in epithelial cells compared to stromal cells (2.8% and 1.4%, respectively). Additionally, estrogen receptor expression is higher in benign phyllodes tumors than in borderline or malignant ones and is inversely related to the mitotic count in stromal cells. This suggests that epithelial expression of estrogen receptors may be related to stromal proliferation. Another study found that estrogen receptor β is present in the stromal cells of fibroadenomas and phyllodes tumors and may be related to smooth muscle differentiation [13], but there is no information regarding the level of expression of this receptor and the mitotic count. In our investigation, there is no information on the effects of endocrine treatment, such as aromatase inhibitors or tamoxifen, on phyllodes tumors. In our case, there was a clinically evident response, allowing for a lumpectomy instead of a mastectomy. However, there is no evidence to suggest that this type of treatment could enable breast-conserving surgeries instead of mastectomies in large phyllodes tumors, similar to the use of neoadjuvant treatments in breast cancer, or if it increases disease-free survival. Given the very low mortality rate of benign phyllodes tumors, this treatment may not affect overall survival, but further research is needed.

This patient had a 10-year history of a lump in surveillance considered to be a “benign fibroadenoma”. A case report by Wang et al. [1] explored the clinical history of a 27 years old female with a phyllodes tumor who previously had a fibroadenoma in the same breast. Sanders et al. [14] also reported a case of a female with a fibroadenoma in surveillance for three years, later diagnosed as a phyllodes tumor. These cases raise the question of whether a benign fibroadenoma could transform into a phyllodes tumor over time.

Additionally, the patient was diagnosed with colloid adenocarcinoma of the lung, a rare neoplasm accounting for less than 1% of lung cancers [15]. To our knowledge, this is the first case report of a patient presenting with both a phyllodes tumor and a colloid adenocarcinoma of the lung, initially suspected to be a metastasis of breast cancer. Given the presence of two rare tumors, a genetic study should be conducted for this patient.

## Conclusions

This case report highlights the complexities in diagnosing and managing phyllodes tumors, particularly given their resemblance to fibroadenomas. Our case is unique as it documents the first known response of a phyllodes tumor to anastrozole, an aromatase inhibitor typically used for endocrine receptor-positive breast cancer. Despite the lack of established evidence supporting the use of endocrine therapy in phyllodes tumors, this case demonstrated a significant clinical response, allowing for less invasive surgery. This finding suggests the potential for endocrine therapy as an adjunct treatment in specific scenarios, though further research is needed to substantiate these observations and explore their implications for disease-free and overall survival. Moreover, the co-occurrence of a phyllodes tumor with a colloid adenocarcinoma of the lung underscores the importance of comprehensive and prompt diagnostic evaluations. The patient’s favorable outcome post-surgery and disease-free status at eight months post-treatment are promising, emphasizing the importance of multidisciplinary team discussions and tailored treatment approaches. Future studies are needed to investigate the role of endocrine therapies in phyllodes tumors and explore the genetic underpinnings that may predispose patients to multiple rare cancers.

## References

[1] Wang Q, Su J, Lei Y (2017). Recurrent malignant phyllodes tumor of the breast: a case report. Medicine (Baltimore).

[2] Ditsatham C, Chongruksut W (2019). Phyllodes tumor of the breast: diagnosis, management and outcome during a 10-year experience. Cancer Manag Res.

[3] Zervoudis S, Xepapadakis G, Psarros N (2019). Management of malignant and borderline phyllodes tumors of the breast: our experience. J Buon.

[4] Zhang T, Feng L, Lian J, Ren WL (2020). Giant benign phyllodes breast tumour with pulmonary nodule mimicking malignancy: a case report. World J Clin Cases.

[5] Mabewa AA, Mushi A, Agapit T, Njile J (2022). Phylloides: uncommon ulcerated breast tumor diagnosed at Singida regional referral hospital. Case report. Ann Med Surg (Lond).

[6] Sars C, Sackey H, Frisell J (2023). Current clinical practice in the management of phyllodes tumors of the breast: an international cross-sectional study among surgeons and oncologists. Breast Cancer Res Treat.

[7] Zhang Y, Kleer CG (2016). Phyllodes tumor of the breast: histopathologic features, differential diagnosis, and molecular/genetic updates. Arch Pathol Lab Med.

[8] Hoda SA, Brogi E, Koerner FC, Rosen PP (2014). Rosen's Breast Pathology, 4e. https://pathology.lwwhealthlibrary.com/book.aspx?bookid=1241.

[9] Ogunbiyi S, Perry A, Jakate K, Simpson J, George R (2019). Phyllodes tumour of the breast and margins: how much is enough. Can J Surg.

[10] Zeng S, Zhang X, Yang D, Wang X, Ren G (2015). Effects of adjuvant radiotherapy on borderline and malignant phyllodes tumors: a systematic review and meta-analysis. Mol Clin Oncol.

[11] Chumsri S, Howes T, Bao T, Sabnis G, Brodie A (2011). Aromatase, aromatase inhibitors, and breast cancer. J Steroid Biochem Mol Biol.

[12] Tse GM, Lee CS, Kung FY, Scolyer RA, Law BK, Lau TS, Putti TC (2002). Hormonal receptors expression in epithelial cells of mammary phyllodes tumors correlates with pathologic grade of the tumor: a multicenter study of 143 cases. Am J Clin Pathol.

[13] Sapino A, Bosco M, Cassoni P (2006). Estrogen receptor-beta is expressed in stromal cells of fibroadenoma and phyllodes tumors of the breast. Mod Pathol.

[14] Sanders LM, Daigle ME, Tortora M, Panasiti R (2015). Transformation of benign fibroadenoma to malignant phyllodes tumor. Acta Radiol Open.

[15] Masai K, Sakurai H, Suzuki S, Asakura K, Nakagawa K, Watanabe S (2016). Clinicopathological features of colloid adenocarcinoma of the lung: a report of six cases. J Surg Oncol.

